# Step into the era of large multimodal models: a pilot study on ChatGPT-4V(ision)’s ability to interpret radiological images

**DOI:** 10.1097/JS9.0000000000001359

**Published:** 2024-03-18

**Authors:** Lingxuan Zhu, Weiming Mou, Yancheng Lai, Jinghong Chen, Shujia Lin, Liling Xu, Junda Lin, Zeji Guo, Tao Yang, Anqi Lin, Chang Qi, Ling Gan, Jian Zhang, Peng Luo

**Affiliations:** a Department of Oncology, Zhujiang Hospital, Southern Medical University, Guangzhou; b Department of Etiology and Carcinogenesis, National Cancer Center/National Clinical Research Center for Cancer/Cancer Hospital, Changping Laboratory, Chinese Academy of Medical Sciences and Peking Union Medical College; c Department of Urology, Shanghai General Hospital, Shanghai Jiao Tong University School of Medicine, Shanghai; d Department of Medical Oncology, National Cancer Center/National Clinical Research Center for Cancer/Cancer Hospital, Chinese Academy of Medical Sciences and Peking Union Medical College, Beijing; e Institute of Logic and Computation, TU Wien, Austria; f Department of Ultrasound Medicine, The First Affiliated Hospital, Fujian Medical University, Fujian; g Department of Ultrasound Medicine, National Regional Medical Center, Binhai Campus of the First Affiliated Hospital, Fujian Medical University, Fuzhou, People’s Republic of China

**Keywords:** artificial intelligence, ChatGPT, generative AI, multimodality, radiology

## Abstract

**Background:**

The introduction of ChatGPT-4V’s ‘Chat with images’ feature represents the beginning of the era of large multimodal models (LMMs), which allows ChatGPT to process and answer questions based on uploaded images. This advancement has the potential to transform how surgical teams utilize radiographic data, as radiological interpretation is crucial for surgical planning and postoperative care. However, a comprehensive evaluation of ChatGPT-4V’s capabilities in interpret radiological images and formulating treatment plans remains to be explored.

**Patients and methods:**

Three types of questions were collected: (1) 87 USMLE-style questions, submitting only the question stems and images without providing options to assess ChatGPT’s diagnostic capability. For questions involving treatment plan formulations, a five-point Likert scale was used to assess ChatGPT’s proposed treatment plan. The 87 questions were then adapted by removing detailed patient history to assess its contribution to diagnosis. The diagnostic performance of ChatGPT-4V was also tested when only medical history was provided. (2) We randomly selected 100 chest radiography from the ChestX-ray8 database to test the ability of ChatGPT-4V to identify abnormal chest radiography. (3) Cases from the ‘Diagnose Please’ section in the Radiology journal were collected to evaluate the performance of ChatGPT-4V in diagnosing complex cases. Three responses were collected for each question.

**Results:**

ChatGPT-4V achieved a diagnostic accuracy of 77.01% for USMLE-style questions. The average score of ChatGPT-4V’s treatment plans was 3.97 (Interquartile Range: 3.33–4.67). Removing detailed patient history dropped the diagnostic accuracy to 19.54% (P<0.0001). ChatGPT-4V achieved an AUC of 0.768 (95% CI: 0.684–0.851) in detecting abnormalities in chest radiography, but could not specify the exact disease due to the lack of detailed patient history. For cases from ‘Diagnose Please’ ChatGPT provided diagnoses consistent with or very similar to the reference answers.

**Conclusion:**

ChatGPT-4V demonstrated an impressive ability to combine patient history with radiological images to make diagnoses and directly design treatment plans based on images, suggesting its potential for future application in clinical practice.

## Introduction

Large-language models (LLMs) represented by ChatGPT have received widespread attention since their launch^1,2^. While not specifically trained for the medical domain, ChatGPT has demonstrated commendable proficiency in various medical subfields, as evidenced by its ability to successfully pass the United States Medical Licensing Examination (USMLE)^3,4^and to help surgeons educate patients about prostate cancer^5^.

The advent of ChatGPT-4V(ision)’s ‘Chat with images’ feature marks the onset of the era of large multimodal models (LMMs), enabling ChatGPT to process and answer questions based on uploaded images^6^. This development could revolutionize how surgical teams interpret and leverage radiographic data. Radiological interpretation plays an indispensable role in the surgical domain, guiding surgeons in preoperative assessments, determining the feasibility of surgical interventions, and influencing postoperative care. While the report by Yang *et al*. mentioned the potential of ChatGPT in interpreting radiographic images through a simple example^7^, there is a lack of systematic evaluation to illustrate the strengths and weaknesses of ChatGPT-4V in medical radiology. Publicly available radiological image resources, such as the USMLE sample question, the ChestX-ray8 database, and clinical cases with radiological images from the Radiology journal, provide a vast repository of data for testing AI models in medical radiology.

In this study, we used image-based questions from the USMLE and its practice exams to test whether ChatGPT-4V could make diagnoses or even determine further treatment plans by reading images in conjunction with patient histories. We also compared the diagnostic accuracy of the ChatGPT-4V model when provided with and without patient histories. We further evaluated the model’s diagnostic accuracy using a public chest radiography image database. Finally, we used cases from the ‘Diagnosis Please’ section of the Radiology journal to assess the model’s performance on complex cases. Our research offers insights into the capabilities and limitations of the multimodal ChatGPT-4V model, illuminating its potential applications in surgical practice and decision-making.

## Methods

### Question acquisition

For USMLE-style questions, we collected test questions with radiological images from the USMLE sample questions available on their official website, as well as from the AMBOSS database—a widely used USMLE preparation question bank^8^. These two resources have been used in previous research to assess whether ChatGPT could successfully navigate the USMLE exam, but questions with images were excluded^4^. We excluded three questions because either the diagnosis for the radiological image was already included in the question stem, or the question was not related to the interpretation of the image. A total of 87 questions were included in subsequent research. In addition to radiological images, these questions also provided detailed information about the patient’s medical history. The questions examine not only the diagnosis but in some cases also the determination of further treatment plans based on the images, so they can simulate clinical scenarios well and have an appropriate level of difficulty.

ChestX-ray8 is a large public dataset of chest radiography images with diagnostic labels, providing only the patients’ ages and sex, without additional clinical information^9^. We randomly selected 100 radiography images from this dataset using a systematic sampling approach consisting of 43 females and 57 males, with a median age of 45.5 and an interquartile range of 33–55 years. Furthermore, to assess ChatGPT’s performance in complex cases, we collected clinical cases from the ‘Diagnosis Please’ section of the Radiology journal. These cases came with complete history information in addition to radiological images, and they are typically less common, feature more complex clinical scenarios, and are challenging to diagnose. Given that ChatGPT-4’s training data is up to January 2022, we selected 16 cases (Cases 302–317) that were published after February 2022. Subsequently, based on the reviewers’ suggestions, we further collected 50 cases (Cases 252–301) that were published before the training data cutoff.

### Collecting and evaluating responses generated by ChatGPT

To collect responses from ChatGPT-4V, we used carefully designed prompts and uploaded the corresponding images from 1^st^ to 14^th^ October 2023 (Supplementary Material, Supplemental Digital Content 1, http://links.lww.com/JS9/C161). Based on the reviewers’ comments, we collected additional responses from 10^th^ December to 15^th^ December 2023. Each question was asked in a different chat window. We used the regenerate function to generate three responses for each question to assess the stability of the model. For images from the ChestX-ray8 dataset and cases from the ‘Diagnosis Please’ section, we informed ChatGPT that it was participating in a diagnostic challenge as a representative of artificial intelligence, thus preventing ChatGPT from stating that it is not a clinical expert and thus refusing to make a diagnosis. We utilized the receiver operating characteristic (ROC) curve to evaluate the performance of ChatGPT-4V in diagnosing abnormalities in chest radiography images. The best cut-off threshold value was determined using the pROC package in R based on the Youden index. For cases from the ‘Diagnosis Please’ section, we provided the medical history associated with the radiological images in the prompts to simulate a scenario in which doctors make diagnoses under real clinical conditions. Due to the limited number of images that can be uploaded, we combined multiple images into a single composite image, titled ‘Diagnosis Please’ and uploaded it. For USMLE-style questions, we submitted only the question stems and images to ChatGPT, without providing options. This approach was based on previous research findings, which indicated that open-ended questions were better at assessing ChatGPT’s grasp of knowledge^10^. It also prevents ChatGPT from being influenced by options or randomly guessing an answer from options, making it more consistent with clinical scenarios. For questions that required formulating subsequent treatment plans based on radiological findings, three reviewers independently rated ChatGPT’s proposed treatment plan using a five-point Likert scale (5 being the best, 1 being the worst). Additionally, we adapted the questions by retaining only sex and age and then submitted them to ChatGPT-4V for a second attempt to evaluate the importance of medical history in making accurate diagnoses in radiology. According to the reviewers’ comments, we also assessed ChatGPT-4V’s performance when only medical history was provided.

### Statistical analysis

For USMLE-style questions, the final accuracy rate was reported using the majority vote method. Specifically, if two out of three responses were correct, we considered that the model correctly answered this question; otherwise, it was recorded as incorrect. Fisher’s exact test was used to compare the diagnostic performance of ChatGPT for different types of imaging modalities and anatomical regions. McNemar’s test was used to compare the diagnostic accuracy of ChatGPT with detailed patient history versus incomplete clinical data. The Intraclass Correlation Coefficient (ICC) was used to assess the consistency among the three reviewers in evaluating the responses related to formulating treatment plans. All analyses were conducted using R (version 4.2.1). Statistical significance was set at *P* less than 0.05.

## Result

### The accuracy of the ChatGPT-4V model in USMLE-style questions

**Figure 1:**
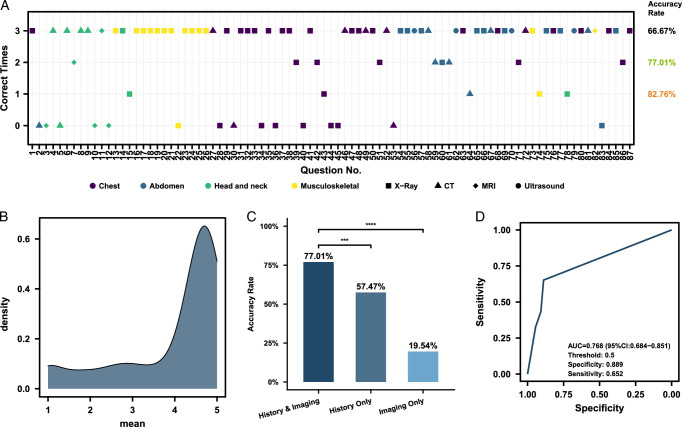
Evaluating the Performance of the Multimodal ChatGPT-4V in Interpreting Radiological Images for Diagnosis and Formulating Treatment Plans. (A) Performance of ChatGPT-4 in USMLE-style questions. The shapes represent different imaging modalities, whereas the colors represent different anatomical regions. The annotations on the right show the accuracy calculated using different criteria. From top to bottom, the criteria are: considering ChatGPT-4 to have answered correctly only if all 3 attempts for a given question are correct; if at least 2 out of the 3 attempts are correct; and if at least 1 of the 3 attempts is correct. (B) Difference in accuracy of multimodal ChatGPT-4V in USMLE-style questions with both image and history, with history only and with image only. ***P<=0.001. ****P<=0.0001 (C) Scores of multimodal ChatGPT-4V in USMLE-style questions involving treatment plan formulation. The score for each question is recorded as the average of the scores given by three reviewers. (D) ROC curve of multimodal ChatGPT-4 in diagnosing the presence of abnormalities in chest radiography from the ChestX-ray8 database. If ChatGPT considers there to be abnormalities, it is scored 1; if it considers there to be no abnormalities, it is scored 0. The sum of the scores from the three attempts was taken to make a comprehensive judgment. AUC, the area under the receiver operating characteristic curve.

A total of 87 questions containing images from different anatomical regions (chest, abdomen, head and neck, and musculoskeletal) and imaging modalities (radiography, computed tomography, MRI, and ultrasound) were included. The questions can be broadly classified into two categories: diagnosis only (51/87) and the development of a treatment plan (36/87). We first evaluated whether ChatGPT-4V could provide accurate diagnostic results for these questions. ChatGPT-4V was able to read and interpret radiological images, analyze the findings, and integrate the patient’s history to make a diagnosis. ChatGPT achieved a diagnostic accuracy of 77.01% when the majority voting method was used to report the final accuracy rate (Fig. 1A). Furthermore, ChatGPT-4V answered correctly at least once in 82.76% of the cases (Fig. 1A). Notably, for some questions, ChatGPT failed to provide the correct answer across three attempts. There was no significant difference in ChatGPT’s diagnostic accuracy across different anatomical regions and imaging modalities (*P* values of 0.15 and 0.28, respectively, Supplementary Table 1, Supplemental Digital Content 2, http://links.lww.com/JS9/C162).

When it comes to developing treatment plans based on the radiological images, ChatGPT-4V demonstrated its ability to combine medical history and imaging data to devise appropriate treatment strategies in most cases, with an average score of 3.97 (Interquartile Range: 3.33–4.67, Fig. 1B; ICC for 3 reviewers: 0.843, 95%CI: 0.792–0.885).

### Evaluating ChatGPT-4V’s performance with limited patient history and only patient history

The original questions were then modified by omitting detailed patient history, retaining only the patient’s sex and age for submission to ChatGPT. We observed that ChatGPT-4V demonstrated significantly higher accuracy in diagnosing images with complete patient histories than images without detailed clinical information (*P*<0.0001, Fig. 1C). We also tested the performance of the ChatGPT-4V in making diagnoses based solely on patient history. The results showed that the accuracy was lower than when providing both detailed medical history and radiographic images (*P*<0.001, Fig. 1C).

### Performance of the ChatGPT-4V model on the ChestX-ray8 dataset

We then evaluated the diagnostic accuracy of the ChatGPT-4V model on images from the ChestX-ray8 chest radiography database. ChatGPT-4V was able to identify abnormalities (such as patchy opacities), but faced challenges in accurately classifying the detected radiographic findings into diagnostic labels. Therefore, we focused our current evaluation on whether ChatGPT-4V could detect the presence of abnormalities in chest radiography. Taking into account the results of three attempts, the ChatGPT-4V model achieved an AUC of 0.768 (95% CI: 0.684–0.851) with a sensitivity of 0.652 and a specificity of 0.889 in determining the presence of abnormalities in the chest radiography (Fig. 1D).

### Performance of the ChatGPT-4V model in the cases from ‘Diagnosis Please’ section

Cases from the ‘Diagnosis Please’ section of radiology journals were used to assess the performance of ChatGPT-4V in analyzing complex cases. ChatGPT demonstrated impressive abilities by methodically analyzing each image in the case, summarizing the findings from the radiographic images, integrating them with the patient’s history, and then proceeding to provide a diagnosis and diagnostic reasoning process (Table 1, Supplementary Table 2, Supplemental Digital Content 3, http://links.lww.com/JS9/C163). In several cases, ChatGPT-4V’s responses aligned perfectly with the reference answers, and in some other cases, they reached concordance at least once. However, due to the complexity of certain cases and the limitations of ChatGPT-4V’s capabilities, it failed to achieve agreement with the reference answer across three attempts for some cases.

**Table 1:** Diagnoses given by the multimodal ChatGPT-4V Model for the cases in the ‘Diagnosis Please’ Section and the reference answers (Case 302–317).

Case	Reference answer	Try 1	Try 2	Try 3
302	Supratentorial Lymphocytic Inflammation with Parenchymal Perivascular Enhancement Responsive to Steroids	Neurocysticercosis	Neuromyelitis Optica Spectrum Disorder	Neuro-Behçet’s Disease
303	Delayed Posthypoxic Leukoencephalopathy	Hypoxic Ischemic Encephalopathy (HIE) or Anoxic Brain Injury	Hypoxic-Ischemic Encephalopathy (HIE)	Hypoxic-Ischemic Encephalopathy
304	Porphyria Cutanea Tarda	Hemochromatosis	Hemochromatosis	Hemochromatosis
305	Loeffler Endocarditis	Löffler endocarditis or eosinophilic myocarditis	Eosinophilic Myocarditis	Löffler endocarditis or Eosinophilic myocarditis
306	Ectopic Thyroid Goiter in the Porta Hepatis	Ectopic thyroid tissue in the porta hepatis	Ectopic thyroid tissue in the porta hepatis	Ectopic thyroid tissue in the porta hepatis
307	Heterotopic Pancreas in Jejunal Mesentery	Mesenteric Fibromatosis (Desmoid Tumor)	Mesenteric Paraganglioma	Pancreatic nonfunctioning neuroendocrine tumor (nonfunctioning pancreatic NET)
308	Van Wyk–Grumbach Syndrome	Van Wyk-Grumbach syndrome	Van Wyk-Grumbach Syndrome	Van Wyk-Grumbach Syndrome
309	Autoimmune Glial Fibrillary Acidic Protein Astrocytopathy	Primary Central Nervous System Lymphoma	Primary Central Nervous System Lymphoma	Primary Central Nervous System Lymphoma
310	Posterior Epidural Migration of a Lumbar Disk Herniation	Lumbar Epidural Abscess	Epidural Abscess	Spinal Anterior Epidural Abscess
311	Generalized Lymphatic Anomaly	Hodgkin lymphoma	Lymphoma	Lymphangioma or Cystic Hygroma.
312	Dercum Disease	Familial Multiple Lipomatosis	Familial Cystic Mastopathy	Familial Multiple Lipomatosis
313	Cerebral Venous Infarct Due to Internal Cerebral Vein Thrombosis in the Setting of COVID-19 Infection	Cerebral Venous Thrombosis (CVT) secondary to a pulmonary condition (possibly an infection or inflammatory condition).	Cerebral Venous Sinus Thrombosis (CVST) with associated brain infarctions and potential pulmonary embolism or diffuse pulmonary pathology.	Metastatic cancer to the brain, most likely from a primary lung malignancy.
314	Cerebral Proliferative Angiopathy	Arteriovenous Malformation	Arteriovenous Malformation	Cerebral Arteriovenous Malformation
315	Cardiac Calcified Amorphous Tumor	Cardiac Myxoma	Cardiac Myxoma	Cardiac Myxoma
316	Progressive Pseudorheumatoid Dysplasia	Mucopolysaccharidosis (MPS)	Mucopolysaccharidosis type IV (Morquio syndrome)	Progressive Pseudorheumatoid Dysplasia
317	Adult-Onset Leukoencephalopathy with Axonal Spheroids and Pigmented Glia	Adult-onset Leukoencephalopathy with Axonal Spheroids and Pigmented Glia	Leukoencephalopathy with vanishing white matter	Frontotemporal Dementia

## Discussion

The release of the multimodal ChatGPT-4V model undoubtedly represents another revolutionary advancement in generative artificial intelligence. In our research, we submitted both images and patient histories to ChatGPT, simulating decision-making scenarios for doctors in clinical practice. We directly tested ChatGPT’s imaging diagnostic capabilities at multiple levels, showcasing the performance of the model. ChatGPT achieved a 77.01% accuracy rate on USMLE-style questions and could provide fairly accurate treatment plans based on radiological images and patient histories. Additionally, ChatGPT can identify abnormalities in chest radiography with an AUC of 0.768 (95% CI: 0.684–0.851) but tends to be conservative in its judgments. Notably, ChatGPT also pointed out the presence of medical implants in the chest radiography, such as central venous catheters and cardiac devices. The performance of the ChatGPT-4V Model is also satisfactory in the ‘Diagnosis Please’ section, exhibiting relatively high abnormality detection capabilities. Ueda *et al*.‘s research used the textual descriptions of radiographic images provided in the ‘Diagnosis Please’ cases to indirectly test ChatGPT’s diagnostic abilities, finding that ChatGPT was able to correctly diagnose approximately half of the 317 cases^11^. Our research used patient histories and radiographic images to directly test ChatGPT-4V’s diagnostic performance, showing relatively promising results. In cases where ChatGPT did not align with the reference answer, the diagnoses it provided are sometimes very similar to the reference diagnoses, and these diseases can be challenging to differentiate based on radiographic images, indicating that while ChatGPT’s overall diagnostic approach is correct, it may still lack the necessary precision in handling details. Despite not always aligning perfectly with reference answers, ChatGPT-4V’s diagnoses could still hold clinical value as they may represent reasonable differential diagnoses.

In summary, despite lacking specific pre-training in radiology, ChatGPT-4V’s performance in analyzing radiological images and combining them with medical histories to make a diagnosis was impressive, indicating exciting potential for future development and application.

As a qualification test for medical practitioners, questions from the USMLE exam have an appropriate level of difficulty. ChatGPT-4V’s excellent performance in these questions proves that it has at least a basic level of competence in the field of radiology. Our study also emphasizes the necessity of combining patient history with radiological findings to make an accurate diagnosis. While ChatGPT can identify some abnormal changes in chest radiography, the absence of patient history makes it difficult to provide a definitive diagnosis. For example, patchy opacities in the lung fields could be due to several reasons, such as pneumonia, pulmonary edema, or other lung conditions^12^. This point is further highlighted when testing with questions that removed detailed patient history, leading to a significant drop in ChatGPT’s diagnostic accuracy. Therefore, if ChestX-ray8 could provide detailed patient history, we believe ChatGPT’s diagnostic performance in this dataset would significantly improve. However, it is important to note that there are still some inaccuracies in the responses of the ChatGPT-4V. For example, the treatment plans provided were not always correct. It also tends to mistake gastric bubbles as abnormalities, thus misdiagnosing normal radiography as abnormal. Furthermore, the model sometimes struggles to detect subtle lesions, possibly due to inadequate relevant images in the training data or the insufficient resolution of uploaded images. However, we believe that if it can be further trained with radiology-related knowledge, the performance of ChatGPT-4V will improve and may even surpass existing artificial intelligence models due to its multimodal advantages.

Our research highlights that the latest version of ChatGPT offers a novel opportunity, extending the scope of artificial intelligence capabilities in clinical practice. Traditional radiological AI models are limited to processing images and structured clinical information. In contrast, multimodal ChatGPT-4V can interpret radiological images and mine information from unstructured medical histories, synthesizing both to arrive at more accurate diagnoses. In its present form, we believe ChatGPT has the potential to serve as an assistive tool for radiologists and clinicians. It has the potential to analyze medical images and patient history, extract key findings, generate differential diagnoses, and suggest possible next steps. ChatGPT-4V also has the potential to serve as a screening tool given the high usage of imaging in surgery and accordingly the delayed radiologist’s interpretation. By providing rapid preliminary reads, ChatGPT-4V could help identify cases that require urgent attention. However, it is important to note that ChatGPT-4V’s interpretations would still need verification by radiologists before informing clinical decisions. Furthermore, the application of radiological artificial intelligence is not limited to the interpretation of images. In our study, AI has already demonstrated its capability to bridge from radiological interpretation to suggesting treatment options. The work that ChatGPT can accomplish goes beyond this. We can envision an ideal scenario where multimodal AI reads a patient’s medical history and auxiliary examination results, and autonomously determines if further radiological exams are needed. Once the examination is complete, AI reads the scan results, combines them with the patient’s medical history, makes a radiological diagnosis, automatically generates a radiology report, and formulates treatment recommendations. AI would then explain the findings of the radiological examination and the subsequent treatment plan to the patient in plain language. How far are we from realizing all of this?

Our research demonstrates the impressive potential of the multimodal ChatGPT-4V in interpreting radiological images, providing insights into the model’s strengths and limitations. The strength of our research is that we designed multiple tasks early in the model’s release to assess ChatGPT-4V’s image interpretation capabilities and explored the importance of integrating patient history to explain radiological images. However, we acknowledge that our study has some limitations. As a pilot study, the sample size for this research is relatively small, especially the number of normal (non-abnormal) radiographic images. Although there are large numerical differences in accuracy between different sub-groups in Supplementary Table 1, Supplemental Digital Content 2, http://links.lww.com/JS9/C162, the statistical analysis does not support the conclusion that there are differences in accuracy between sub-groups. Since some sub-groups have relatively small sample sizes, future larger-scale studies will help determine whether ChatGPT-4V’s performance differs across imaging modalities and anatomical sites. Due to the restricted access of ChatGPT-4V and the lack of an API for it at the time of our research, we were unable to test ChatGPT-4V’s performance on the entire ChestX-ray8 dataset. In clinical practice, when reading computed tomography and MRI images, it is necessary to adjust different slices and imaging parameters to obtain various images for diagnosis. However, given ChatGPT’s current limitations on the number of images that can be uploaded, we could only select representative slices for diagnosis. With technological advancements, allowing more images to be uploaded would help improve ChatGPT’s performance in interpreting radiology images. Additionally, the auxiliary examination information provided was not comprehensive. Future studies should consider combining pathology, laboratory tests, radiology, and other auxiliary examinations, using patients’ complete clinical data to test the performance of multimodal AI in diagnosis and formulating treatment plans, which is expected to yield better results. More importantly, fine-tuning needs to be performed for specific radiology tasks to improve diagnostic performance. We believe that further exploration of the development trends of such models is warranted in the future, along with a more comprehensive investigation to explore the application value of LMMs in surgery and their impact on clinical practice. We are confident that with technological advancements, LMMs specifically trained for medicine will play a pivotal role in future clinical practice.

## Ethical approval

Not Applicable.

## Consent

Not Applicable.

## Sources of funding

Not applicable.

## Author contribution

L.Z., W.M.: conceptualization, investigation, writing – original draft, methodology, literature review, writing – review and Editing, and visualization; Y.L.: investigation, writing – original draft, methodology, literature review, writing – review and editing; J.C., S.L. and A.L.: conceptualization, writing – review and editing, and literature review. L.X., J.L., Z.G.: investigation, writing –review and editing; T.Y.: writing –review and editing, and literature review; C.Q.: resources and writing – review and editing; L.G.n: conceptualization, writing - review and editing, methodology, supervision; J.Z. and P.L.: conceptualization, literature review, project administration, supervision, resources, writing – review and editing; all authors read and approved the final manuscript.

## Conflicts of interest disclosure

The authors declare that the research was conducted in the absence of any commercial or financial relationships that could be construed as a potential conflict of interest.

## Research registration unique identifying number (UIN)

Not Applicable.

## Guarantor

Lingxuan Zhu, Weiming Mou, Peng Luo, and Jian Zhang

## Data availability statement

The prompts used in this study can be found in the supplementary material. Data generated or analyzed during the study are available from the corresponding author by reasonable request.

## Provenance and peer review

Not commissioned, externally peer-reviewed.

## Supplementary Material

SUPPLEMENTARY MATERIAL
