# Supplementary material for: Step into the era of large multimodal models: a pilot study on ChatGPT-4V(ision)’s ability to interpret radiological images
Source: Int J Surg. 2024 Mar 18;110(7):4096–102. doi: 10.1097/JS9.0000000000001359 (PMC11254196; doi:10.1097/JS9.0000000000001359)
Supplement: SUPPLEMENTARY MATERIAL [file js9-110-4096-s002.docx]

**Supplementary Table 1.** Diagnostic Accuracy of the Multimodal ChatGPT-4V Model for Different Anatomical Regions and Imaging Modalities in USMLE-style Questions.

| Category | Number of questions | Number of correct answers | P value |
| --- | --- | --- | --- |
| **Different Anatomical Regions** | | | 0.153 |
| Chest | 37 | 28 (75.7%) |  |
| Abdomen | 22 | 19 (86.4%) |  |
| Head and neck | 13 | 7 (53.8%) |  |
| Musculoskeletal | 15 | 13 (96.7%) |  |
| **Different Imaging Modalities** | | | 0.281 |
| X-Ray | 58 | 46 (79.3%) |  |
| CT | 19 | 14 (73.7%) |  |
| MRI | 6 | 3 (50.0%) |  |
| Ultrasound | 4 | 4 (100.0%) |  |
